# Performance Evaluation of Hybrid Crowdsensing and Fixed Sensor Systems for Event Detection in Urban Environments †

**DOI:** 10.3390/s21175880

**Published:** 2021-08-31

**Authors:** Matthias Hirth, Michael Seufert, Stanislav Lange, Markus Meixner, Phuoc Tran-Gia

**Affiliations:** 1 User-Centric Analysis of Multimedia Data Group, TU Ilmenau, 98693 Ilmenau, Germany; 2 Chair of Communication Networks, University of Würzburg, 97070 Würzburg, Germany; michael.seufert@uni-wuerzburg.de (M.S.); markus.meixner@uni-wuerzburg.de (M.M.); trangia@informatik.uni-wuerzburg.de (P.T.-G.); 3 Department of Information Security and Communication Technology, NTNU, 7491 Trondheim, Norway; stanislav.lange@ntnu.no

**Keywords:** crowdsensing, event detection, detection time simulation, performance analysis

## Abstract

Crowdsensing offers a cost-effective way to collect large amounts of environmental sensor data; however, the spatial distribution of crowdsensing sensors can hardly be influenced, as the participants carry the sensors, and, additionally, the quality of the crowdsensed data can vary significantly. Hybrid systems that use mobile users in conjunction with fixed sensors might help to overcome these limitations, as such systems allow assessing the quality of the submitted crowdsensed data and provide sensor values where no crowdsensing data are typically available. In this work, we first used a simulation study to analyze a simple crowdsensing system concerning the detection performance of spatial events to highlight the potential and limitations of a pure crowdsourcing system. The results indicate that even if only a small share of inhabitants participate in crowdsensing, events that have locations correlated with the population density can be easily and quickly detected using such a system. On the contrary, events with uniformly randomly distributed locations are much harder to detect using a simple crowdsensing-based approach. A second evaluation shows that hybrid systems improve the detection probability and time. Finally, we illustrate how to compute the minimum number of fixed sensors for the given detection time thresholds in our exemplary scenario.

## 1. Introduction

For decades, people have been collecting sensor measurements in urban areas to derive environmental models or to adapt their behavior to changing situations, such as traffic routing concerning the current traffic volumes. In the past, the processes of data collection, data analysis, and deduction of models or action guidelines were time-consuming, and the overall coverage of the sensing information was somewhat limited due to the required number of dedicated sensing equipment; however, the rise of novel concepts such as smart cities creates an increasing demand for fine-grained and up-to-date environmental information, which cannot be fulfilled with traditional approaches that solely build on a small number of highly specialized (offline) sensing equipment.

One possibility to tackle this challenge is the usage of a large number of Internet of Things (IoT)-based sensing nodes. Recently, many vendors started to offer cheap hardware boards that combine Internet connectivity, low power consumption, and simple programmability. Again, these boards can be used as a basis for customized sensing nodes that continuously deliver real-time environmental data. Another option to collect large amounts of environmental data is mobile crowdsensing (MCS). With the still increasing number of smartphones, smart devices, and wearables, many people carry a diverse set of sensory equipment, including, for example, microphones, cameras, brightness sensors, and gyroscopes. Due to the connectivity features of the devices, the sensor information can be made available in almost real-time and can often be further combined with location information, e.g., based on the devices’ GPS receiver. MCS tries to leverage this source of sensing data by directly involving people in environmental data collection. Primarily due to the low investment costs and no need for additional sensor hardware deployment, MCS is a promising source for sensor information in smart cities.

One major drawback of MCS is the missing control of the sensor nodes. The reliability and accuracy of sensors built into smart devices can vary, and so the sensor’s position (e.g., handheld, inside a bag, or a backpack) is unreliable. Further, the spatial distribution of the sensor nodes is often hard to influence. The sensor nodes, i.e., the smart devices, are carried by the participating citizens, and the density of the sensory network is consequently highly correlated with the population density. Considering the daily routines of the MCS participants, e.g., going to work in the morning and returning home at night, the population density and the geographical density of the sensor network change even during the day. Concerning this limitation, two questions arise: how good the actual sensor coverage of an MCS approach is and whether MCS can reliably detect spatial events.

In this work, we address this question using a simulative evaluation of a real-life scenario in which inhabitants of a small city contribute to an MCS system to detect different types of spatial events that can be correlated or uncorrelated with the density of people. We analyze the detection probability for all event types, i.e., the share of events detected by the MCS users and the detection time, i.e., the time between the event’s appearance and its detection. The traffic infrastructure in our example uses OpenStreetMap data for the city of Würzburg, Germany, and the movement patterns of the MCS participants are generated using the Simulation of Urban Mobility (SUMO) [1]. Our first results show that even if only 1% of the 125,000 inhabitants of the city contributes to the MCS system, correlated events can be detected with a very high probability and within a short time after their occurrence. In contrast, uncorrelated events are harder to detect using an MCS-based approach, and only about 30% of them can be detected in a reasonable amount of time [2]. These results further confirm the need to consider additional means to increase the sensor coverage and decrease event detection time. One option to improve current systems is using hybrid systems of MCS with additional fixed sensors that can help assess the quality of MCS data better and fill areas with a low density of crowdworkers. Thereby, trade-offs need to be made between fixed sensors’ costs and the resulting improvements in sensor coverage and event detection performance.

This work extends our previous publication [2] with the introduction and evaluation of such hybrid systems that employ fixed sensors in addition to MCS. The evaluation results show that fixed sensors can further increase the detection performance; however, the optimal placement of fixed sensors is not trivial as it depends on the event type, the movements of the mobile sensors, and the available budget for buying, installing, operating, and maintaining fixed sensors. Nevertheless, the results can serve as a baseline and benchmark for the performance evaluation of more sophisticated placement approaches that are expected to bring additional benefits to MCS.

The remainder of the paper is structured as follows. Section 2 reviews related work and puts our work in context. Section 3 details on the methodology, including the generation of the events, the mobility model used for the citizens, and the event detection. Section 4 presents and discusses the performance of a purely crowdsensing-based system for the detection time and detection probability of events. Section 5 illustrates the benefits of a combined approach of MCS and fixed sensors. Section 6 discusses the findings and points out directions for future research. Section 7 concludes our paper.

## 2. Related Work

In this section, we outline general concepts that are important for MCS. These include use cases, goal functions of platform providers, system architectures, and incentives to increase user participation. Additionally, we present related work that deals with MCS, particularly considering location-specific tasks, spatial coverage, and user mobility. Furthermore, we provide an overview of hybrid systems that combine sensed data from fixed sensors and mobile users.

### 2.1. Crowdsensing Systems

The widespread availability of smartphones, which are equipped with different sensors and cameras, has paved the way for large-scale MCS and a multitude of crowdsensing applications [3,4]. These include use cases such as temperature and traffic monitoring [5], detection of traffic regulators [6], WiFi localization [7], as well as WiFi characterization [8].

Thereby, MCS systems have to cope efficiently with a large amount of sensor data and a high frequency of information exchange. Thus, several architectural frameworks have been proposed [9,10], which standardize the common steps of sensing, transmission, aggregation, processing, and forwarding to applications. Additionally, there are protocols [11] and applications [7], which reduce the overhead in terms of energy consumption of MCS to lower the barrier for end-user participation.

There are two main goals for MCS service providers, first, minimizing the cost for sensing, and second, maintaining high quality and reliable data [5]. In addition, high user participation is required for keeping sensed information up-to-date. Several research initiatives have already been dealing with the topic of incentives [12,13,14], primarily focusing on monetary incentives, which can be tuned to favor honest reports while minimizing expenditures for the provider. Such techniques include reverse auction approaches [15], Stackelberg game models [16], reputation systems that quantify users’ trustworthiness [17], as well as systems for estimating and utilizing the users’ expertise [18]. Recently, privacy concerns are additionally addressed by incentive mechanisms to increase data trustworthiness, e.g., [19,20]. Further, different methodologies, such as game theory, are applied to optimize MCS systems [21] in general; however, we assume a simplified setting in this work, i.e., users provide honest reports, and we do not consider energy consumption and privacy. This simplified setting allows us to derive generalizable results that can be used as benchmarks for more sophisticated real-world settings.

### 2.2. Mobility and Location Awareness

Several works already tackled the challenges of location-specific tasks, i.e., sensing events, which occur at specific locations and whose detection requires the presence of a nearby participant. Those works were summarized in several surveys [22,23,24], and as such, only the most relevant works are discussed further.

While in our work, users do not stray from their regular path to detect events, the authors of [25] propose the notion of a time budget, which can be spent on detours for MCS tasks; however, they note that the corresponding optimization problems for achieving minimal costs are NP-hard and propose heuristic and approximation algorithms to cope with large-scale problem instances. Another particular issue, which was addressed in [26], is the location uncertainty that arises from users who hide their location due to privacy concerns.

As an alternative to pure MCS, hybrid sensor deployments are discussed in [27]. The authors add readings from fixed sensors and cameras to the crowdsensed data in order to increase the performance in terms of precision and coverage. To fully leverage the benefits of such hybrid systems as well as systems that feature only fixed sensors, an optimization of the spatial placement of the fixed sensor’s needs is necessary [28,29] and a suitable notion of coverage should be chosen [30]. Concerning our particular scenario, this means less-frequented city areas would be candidates for sensor locations to enable quick event detection despite a low population density.

Finally, particular MCS services might not need to activate all potential participants to meet their constraints. In these cases, the MCS provider might choose to recruit only a subset of users to minimize the costs. The selection constitutes an optimization problem, and it was shown in simulation studies [31] as well as case studies [32] that algorithmic user selection strategies could significantly reduce the payments while maintaining high service quality. With respect to our scenario, this means such techniques could also be implemented to reduce costs during busy hours when the population density in urban areas is typically higher than necessary to achieve almost instantaneous detection of events. Moreover, exchanging and aggregating data locally before sending them to the service provider based on social interaction between humans can be used to reduce network overhead [33]. Recently, it was also proposed to utilize deep learning to increase the robustness of mobile MCS in terms of sensing platform utility and data accuracy [34].

## 3. Methodology

In this work, we focus on the event detection scenario for the performance evaluation of MCS. This means that events appear at random times and locations on a map and have to be detected as fast as possible by the sensors. In this scenario, when ignoring the shielding caused by obstacles, a regular placement of fixed sensors could cover the whole map, which would lead to an immediate detection of all occurring events; however, depending on the detection range of the sensors, a large number of fixed sensors would be required, which causes high capital and operational expenditures. To reduce these costs, we investigate the potential savings and trade-offs of MCS in this work.

We assume that users perform opportunistic crowdsensing, which means that they can freely move on the map and passively sense their environment. In particular, users are not instructed to move to or sense a specific area. Moreover, they might even be completely unaware of the sensing, e.g., if the sensing is implemented as a background process on their smartphone. We consider these users as mobile sensors, which, similar to fixed sensors, have a given detection range. The coverage of the mobile sensors is thus determined by the activity and mobility of the users, such that there is a probabilistic availability of measurements from mobile sensors in terms of time and location. We conduct the performance evaluation based on a discrete event simulation, which is described in full detail in the following.

### 3.1. Event Appearance

In the performance evaluation, we simulated the occurrence of 1,000,000 events. The time of appearance of each event is independently and uniformly distributed over one day and the location of each event is determined randomly according to one of three methods.

(1) First, we consider *uncorrelated events*, which means that their location of appearance is uncorrelated to the density of people. These events are inspired by events in the real world, such as rain or lightning strikes, which are also completely or at least to a large extend uncorrelated to the population density. In this case, the event location is uniformly distributed over the map. (2) Second, events might also be correlated to the density of people. As a real-world analogon, consider here, for example, accidents or traffic jams that occur more frequently at locations that more people visit. To realistically model such events in the simulation, the location of *correlated events* is distributed identically to the density of people. (3) Finally, we also consider *partially correlated events* that depend on people only to some extent. An example of such events is a fire, which could be caused by humans (correlated) or by sunlight (uncorrelated). Partially correlated events are modeled by introducing a percentage *p*, such that *p*% of the events are correlated events, i.e., their location is distributed according to population density, and (100 − *p*)% are uncorrelated events, i.e., their location is uniformly distributed. These three types of events can approximate a huge set of real-world events, such that our simulative performance evaluation can provide general theoretical results for MCS-based event detection.

### 3.2. User Mobility

We employ the free and open traffic simulation suite SUMO (Simulation of Urban Mobility) [1,35], which was implemented by Deutsches Zentrum für Luft- und Raumfahrt (DLR, German Aerospace Center) to generate synthetic, but realistic movements of users for our performance evaluation. SUMO allows the modeling of intermodal traffic systems, including road vehicles, public transport, and pedestrians. A key feature of the highly customizable tool is that it can create mobility traces for arbitrary cities (e.g., based on OpenStreetMap data) and purposes (e.g., traffic light control, and emission calculation).

We our MCS event detection scenario took place in the small city of Würzburg, Germany, which has a population of 130,500 [36] people and generated a pedestrian mobility trace with SUMO for a whole day. For this, a map of Würzburg of size 8.75 km × 6.05 km was obtained from OpenStreetMap and imported into SUMO as a road network. Every second, the mobility simulation spawns one new pedestrian at a random location on the map. Then, the pedestrian walks through the city to a random destination and vanishes. For each of these trips, two edges of the SUMO network, i.e., roads on the Würzburg map, are selected uniformly random as the start and end of the walk, constrained by a maximum walking distance of 2 km. SUMO performs a fastest-path routing using Dijkstra’s algorithm [37] to determine the intermediate roads. According to the trip definition and using a pedestrian mobility model with default parameters [38] (e.g., maximum speed 5.4 km/h), the position of the pedestrian is updated every second and added to the mobility trace file. In total, the resulting mobility traces include walks on the Würzburg map for a period of 30 h, i.e., a whole day plus some margins before and after the evaluated time frame.

**Figure 1:**
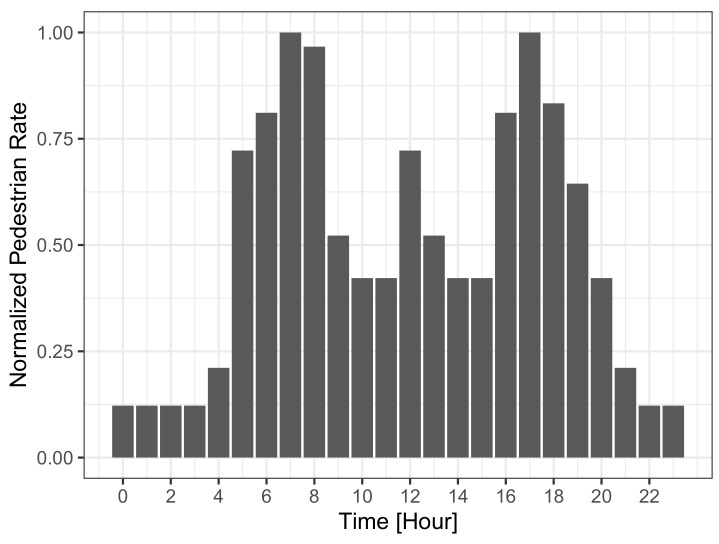
Normalized pedestrian rate with respect to peak rate. Adapted from the typical hourly vehicle volume of streets [39].

**Figure 2:**
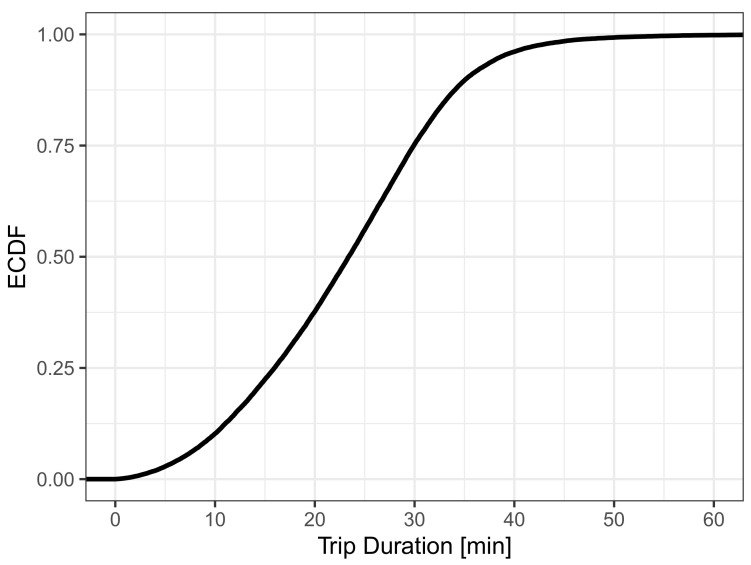
Distribution of trip duration.

To model diurnal activity patterns, we thin out the spawning of pedestrians based on the typical hourly vehicle volume on streets [39]. Figure 1 depicts the normalized traffic volume with respect to peak rate, which then also resembles how the pedestrian rate was thinned out. It can be seen that the peak rate of one new pedestrian per second is reached only for hours, which typically have peak traffic volume. For the other hours, pedestrians can only spawn with a probability corresponding to the relative traffic volume. After thinning, the mobility trace contains the locations of 43,355 unique pedestrians that perform walks with an average trip length of 1660.77 m. Figure 2 presents the empirical cumulative distribution function (ECDF) of the trip duration. Apart from slight deviations for very short and very long trips, we observe an almost uniform distribution having an average walking duration of 1387.82 s (ca. 23 min). This means, following Little’s law, that on average $L=\lambda \;\cdot \;W=1^{\;1\;/\;s}\;\cdot \;1387.82\;s\approx 1388$ pedestrians participate in crowdsensing during peak hours, which is roughly 1% of the population of the simulated city.

### 3.3. Event Detection

We consider every participating pedestrian as a mobile sensor that can detect events. As described above, we focus our evaluation scenario only on events such as fire, rain, and traffic incidents that can easily be recognized and reported by all humans via smartphones. Note that we explicitly leave out events that require dedicated sensing equipment such as air pollution; however, as this would only decrease the MCS population, the modeling could be also adjusted for such events in the future.

We further assume that all events that can be detected by the participating pedestrians, i.e., the mobile sensors, could also be detected by fixed sensors; however, in practice, this would require specialized sensors for each type of event. For example, fires could be detected by infrared cameras or simple smoke detectors. Rain and other weather effects are monitored by official stations or, more recently, also with private IoT devices. Inner-city and highway traffic are often monitored using surveillance cameras or dedicated sensors built directly into the roads. Alternatively, applications such as Google Maps or navigation software use the movement patterns and the current users’ mobility information, i.e., position, velocity, and density of users, to estimate traffic density or detect traffic incidents. To limit the parameter set of our analysis and achieve a general performance evaluation of MCS, we do not differentiate between the actual types of events, such as fire, rain, or traffic incidents, and do not consider the different types of required sensors, their costs, and detection ranges. In contrast, we assume a hypothetical generic sensor or crowdsensing user that is able to detect any occurring event as long as the user or the sensor is within the detection range. Consequently, the detection range of the sensors has to be modeled. For this, we divide the map into a regular grid of small cells of width 50 m. Moreover, we use the simple assumption that an event can be detected if a sensor is in the same cell as the event, and we compute the detection time of an event as the time from the appearance of an event until a sensor covers the event cell. Note that the detection time is 0 if a fixed or mobile sensor is co-located in the same cell during the appearance of an event.

## 4. Crowdsensing Results

**Figure 3:**
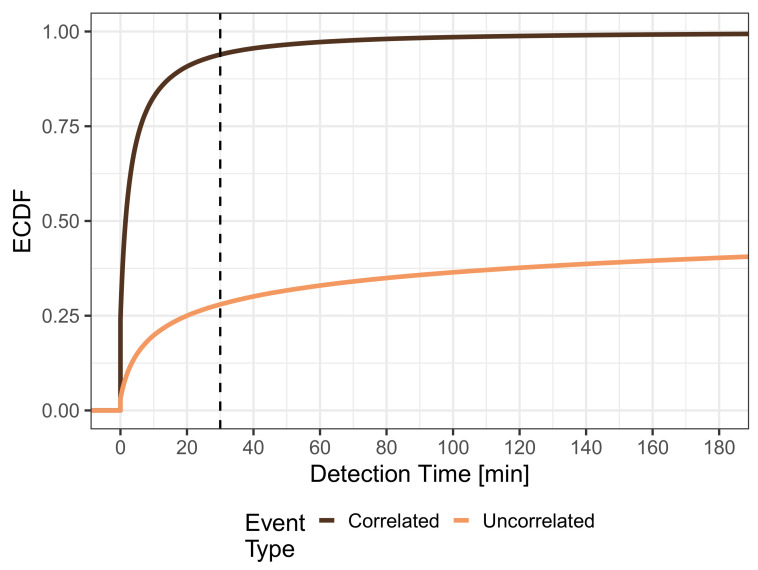
Distribution of detection time for uniform and population based events.

We quantify the performance of MCS to detect events in terms of detection time and detection probability. First, we investigate the ECDF of the detection time, which is depicted in Figure 3, and look at the distribution of detection times for uncorrelated events with a uniformly distributed location, which is given by the light curve. The curve shows that around 3% of the events have a detection time of 0, which means they are detected immediately because a mobile sensor is already present when an event appears. The ECDF shows a fast increase in the first quartile, which eventually slows down. It can be seen that 59.73% of the events have a detection time larger than 180 min or are never detected. This finding was expected given that the map has areas where only a few roads are located or no roads at all. Consequently, there is a significantly lower number of potential visitors in these areas, making it difficult or impossible to detect events that appear in these cells using MCS. In particular, when inspecting the mobility trace, we see about 49% of all cells were not visited by any mobile sensor, which is well aligned with these results. The dark curve represents the ECDF of the detection time for correlated event locations, which are distributed identically to the population density of the mobile trace. It can be seen that 24.18% of the events can be detected immediately, and generally, the events are detected much faster. The median detection time is 91 s and only very few events (0.07%) have a detection time larger than 180 min or are never detected. Further, these results are not surprising given that population density and event density perfectly match. Consequently, events are more likely to occur in cells that people often visit, and thus, MCS is well suited to detect such events quickly.

In the real world, almost no event will remain apparent or relevant for an infinite amount of time. Instead, events can disappear after some time, e.g., a traffic jam dissolves or occasional rain turns into a heavy shower. These events have to be detected within a particular time to provide helpful information, e.g., until the next traffic report or weather forecast on a radio channel. In practice, this maximum detection time is determined by the event type; however, by just inspecting the shapes of the distributions in Figure 3, we can already clearly see that the detection of uncorrelated events is much more sensitive to setting a maximum detection time than the detection of correlated events.

**Figure 4:**
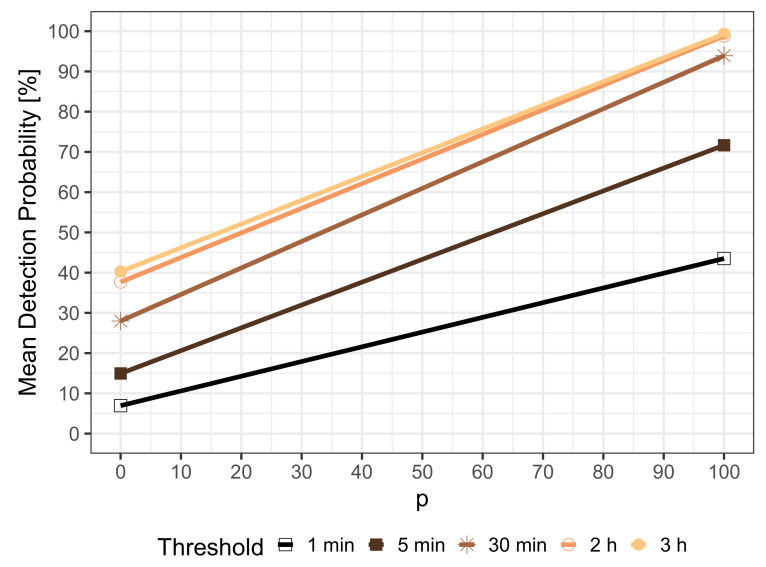
Detection probabilities for partially correlated events and different maximum detection time thresholds.

To gain more insights, we investigate the trade-offs between the detection probabilities and different maximum detection time thresholds in Figure 4. The percentage *p* of correlated events is plotted on the x-axis, meaning that (100 − *p*)% of the events are uncorrelated, and differently colored lines represent different maximum detection time thresholds. Partially correlated events are linearly combined from the set of uncorrelated and correlated events by design, and single events appear independently and are detected independently; therefore, a linear increase can be observed from the detection probability of uncorrelated events $dp_{uncorr}$ in case of p=0 (only uncorrelated events) to the detection probability of correlated events $dp_{corr}$ for p=100 (only correlated events). This means that we can compute the detection probability $dp_{p}$ of partially correlated events with percentage *p* as

$$dp_{p}=p\%\;\cdot \;dp_{corr}+\left(100-p\right)\%\;\cdot \;dp_{uncorr}.$$

Considering a maximum detection time threshold of 3 h, the resulting detection probabilities are 42.03% for uncorrelated events and 99.31% for correlated events, which can be seen at the right margin of Figure 3. When lowering the maximum detection time threshold down to 1 min to focus only on very fast event detection, the detection probabilities decrease. Nevertheless, 6.94% of uncorrelated events and 43.51% of correlated events can be detected within one minute. This clearly indicates that the threshold for the maximum detection time, which in practice is defined by the type of detected event, has a huge impact on the performance of MCS. A 30 min threshold is a good compromise of low detection times and high detection probabilities, which range from 27.98% for p=0 (only uncorrelated events) to 93.91% for p=100 (only correlated events). Thus, in the rest of this section, we will assume a maximum detection time of 30 min. This means an event is considered not detected or missed if the detection time is larger than 30 min.

**Figure 5:**
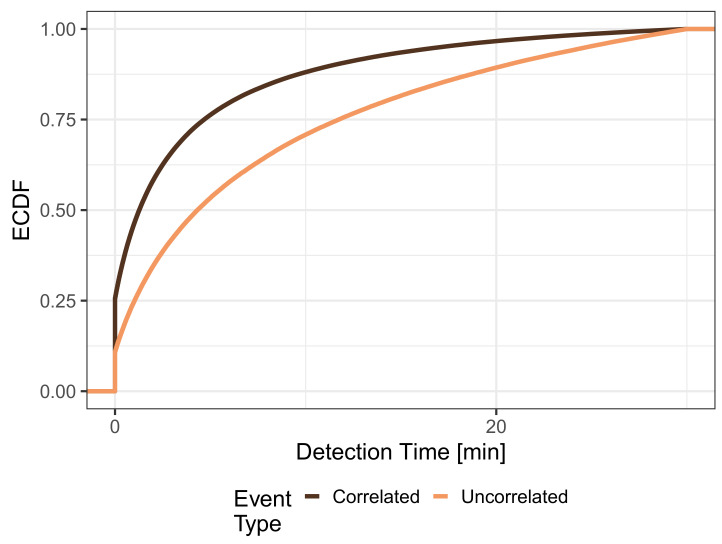
Distribution of detection time for events with maximum detection time of 30 min.

Figure 5 depicts the detection time distributions when considering only events, which have a maximum detection time of 30 min. Thus, the figure shows the detection time distribution from Figure 3 truncated at 30 min. The plot shows that the detection times are still shorter for correlated events, which is expected, but the shapes of the ECDFs have become more similar. In this case, the respective probabilities of immediate detection are 11.0% for uncorrelated and 25.7% for correlated events, respectively.

**Figure 6:**
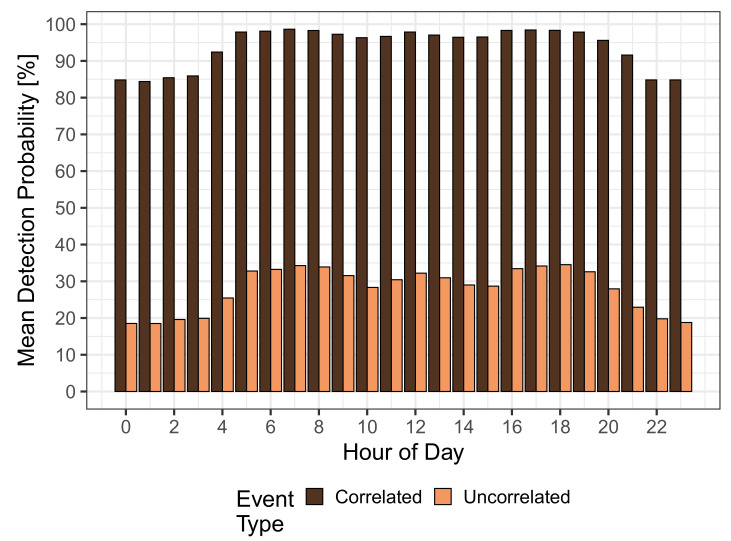
Hourly detection probability.

As we have already noted above, the detection probability is heavily influenced by the maximum detection time of 30 min. When looking at the hourly detection probability in Figure 6, i.e., the detection probability of an event that appeared during a particular hour of the day, two interesting observations become evident. The first observation, as also indicated by Figure 3, is that correlated events (dark curve) have high detection probabilities and reach a maximum of 98.6%. Even the minimum detection probability of 84.4% during nighttime is considerably high and confirms that MCS can be applied to detect such events. When considering uncorrelated events (light curve), detection probabilities are much smaller and range between 18.5% and 34.5%. Thus, for these events, MCS shows a relatively poor event detection performance and, therefore, has to be complemented by fixed sensors to achieve a serviceable detection probability. The second observation is that the shape of the hourly detection probability resembles the MCS participation in Figure 1. This shape is more pronounced for uncorrelated events because, for correlated events, it is superimposed with the generally high detection probability in this case. Nevertheless, this shows that increasing the participation in MCS results in higher detection probabilities for all kinds of events.

**Figure 7:**
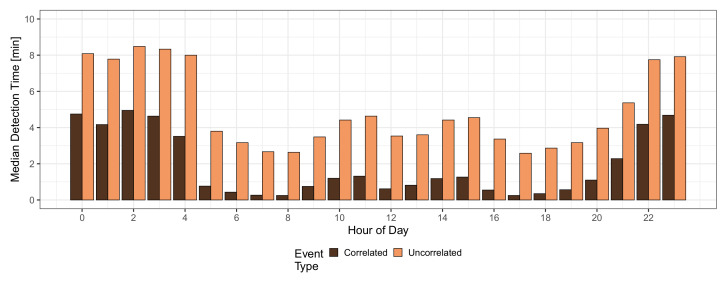
Hourly median detection time.

Next, the hourly median detection time is investigated in Figure 7, i.e., the median detection time of an event that appeared during that hour of the day. The dark bars depict the median detection time for uncorrelated events, and it is visible that the shape resembles the inverse of the crowdsensing participation in Figure 1. It can be seen that the highest median detection times of up to 8.5 min occur during nighttime, as expected when only a few people are walking on the streets and actively sensing. In contrast, for hours with high MCS participation, the median detection times decrease down to 2.6 min at 7 a.m., 8 a.m., and 5 p.m. The corresponding hourly median detection time for correlated events is depicted as light bars. The trend of the median detection time is similar to the uncorrelated events, but the absolute values are much lower. At peak hours, many events are detected immediately, resulting in a median detection time of 16 s. The highest median detection time is 5.0 min at 2 a.m., which is still very low considering the low participation in MCS in the middle of the night. Although these numbers seem small, it has to be remembered that the detection time distributions have a long tail, which could be seen in Figure 3. Thus, although low median detection times are reported here, many events face much larger detection times or cannot be detected at all. Nevertheless, MCS can detect most events quickly, which again confirms that MCS is a promising approach for detecting correlated events.

## 5. Hybrid System of Crowdsensing and Fixed Sensors

Pure MCS has some limitations, especially concerning the detection of uncorrelated events. Consequently, additional means have to be considered. Two possibilities are to add fixed sensors in a hybrid crowdsensing system or to recruit dedicated participants to cover spatial and temporal areas with a low density of people, which is called active crowdsensing.

In a hybrid crowdsensing system, fixed sensors can be placed inside a cell. They will immediately detect all events that appear in the cell, and presumably, the detection will be perfectly reliable for a very long time. Fixed sensors should best be deployed in cells into which people never or rarely move; however, they introduce CAPEX and OPEX for purchase, installation, operation, and maintenance, limiting the number of sensors deployed in such a hybrid system. Moreover, once deployed, it is typically expensive to change the cell of a sensor, which reduces the system’s flexibility.

In active crowdsensing, additional participants are recruited to sense cells with a low density of people or wherever sensor readings are additionally needed. In contrast to fixed sensors, active crowdsensing typically does not involve CAPEX, and crowdworkers can be dynamically instructed about their sensing task, such that this system shows very high flexibility; however, it involves costs to incentivize crowdworkers depending on the active selection strategy, which might consider different aspects, such as availability, mobility, and reliability of crowdworkers, and to reimburse their expenses, such as travel costs. If the sensing budget is exceeded, no more participants can be actively recruited, and the coverage of the active crowdsensing system will decrease to pure MCS.

When deciding between the two alternatives, there are trade-offs whether the sensing budget should be better spent for fixed sensors with high reliability and longevity but low flexibility or for the active recruitment of crowdworkers, which have increased flexibility of data collection but might be less available or reliable. Obviously, in that sense, a hybrid active crowdsensing system can also be implemented consisting of both fixed sensors and additionally recruited crowdworkers.

In this work, as a start, we focus on a hybrid crowdsensing system, in which the detection is improved by adding fixed sensors only. The other option of active crowdsensing can be better evaluated in future works using the performance of this hybrid crowdsensing system as a baseline. In this section, two evaluations of hybrid crowdsensing systems are presented. First, the number of fixed sensors is limited, e.g., due to cost constraints, and the detection performance is studied. Second, the detection performance is predefined, and the needed amount of fixed sensors will be determined.

**Figure 8:**
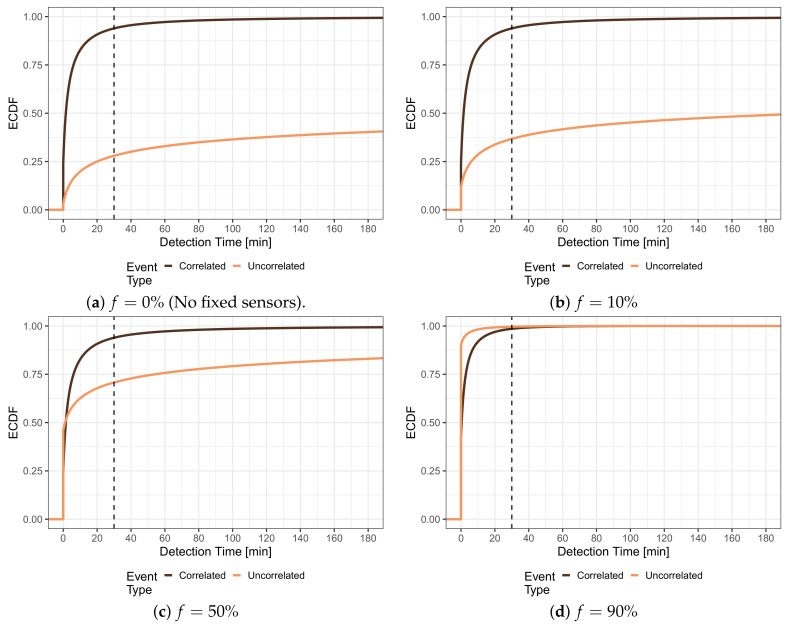
Distribution of detection time for uniform and population-based events in a hybrid system with fixed sensors. The fixed sensors are placed according to the global density of people, i.e., they are placed in the *f*% of cells, which have the lowest density of people.

The numbers presented in the following will consider the expenditures for adding additional fixed sensors to a pure crowdsensing-based system; however, the results can be equivalently applied considering the savings when adding MCS to a system, in which a fixed sensor was deployed in every cell. Figure 8 studies the impact of the number of fixed sensors on the detection times. The share *f* indicates the share of cells, which were equipped with fixed sensors, and thus, can be seen as the parameter, which defines the expenditures for the fixed sensors. The fixed *sensors have been deployed in the f% of cells with the lowest density of people*, and thus, should boost the detection, especially for uncorrelated events. The detection of these uncorrelated events suffered because many cells (49%) were never visited by any person. Figure 8a shows the distribution of detection times for pure MCS, i.e., without any fixed sensors (f=0%), cf. Figure 3, as a reference. Figure 8b illustrates the situation when fixed sensors are distributed to f=10% of the cells. This means that fixed sensors were only placed cells that no person ever visited. It can be observed from the light curve that the share of uncorrelated events that are detected immediately increased from 3% for f=0% to 11.61%. Further, the share of events having a detection time larger than 180 min or not being detected at all decreased from 59.73% for f=0% to 50.09%. The cumulative distribution function is generally shifted to the left compared to the reference, which means that the detection times generally shorten. These effects become stronger when more vacant cells are equipped with fixed sensors. In Figure 8c, half of the cells, and thus all cells that were never visited by any person (49%, c.f. Section 4), contain a fixed sensor. Consequently, the share of immediately detected uncorrelated events reaches 45.97%. When f=90% of the cells contain a fixed sensor, which is depicted in Figure 8d, the detection times of uncorrelated events further decrease, as 90.36% all of these events are immediately detected by a sensor.

It can be seen from Figure 8 that the detection of correlated events (dark curve) is much less affected by adding fixed sensors to cells, which are never visited by any person, because also no correlated events will appear in those cells. Thus, the distributions shown in Figure 8b,c resemble the reference of Figure 8a. Nevertheless, a small decrease in the overall detection times can be observed for Figure 8d. For f>51%, fixed sensors are also deployed in cells that are visited by people, and, thus, the sensors also start detecting correlated events. The positive effect can be seen in Figure 8d from the increase in immediate detected correlated events, from 24.18% for f=0%,f=10%, and f=50% up to around 41.79% for f=90%. Additionally, the detection time distribution is also shifted to the left, i.e., towards shorter detection times.

**Figure 9:**
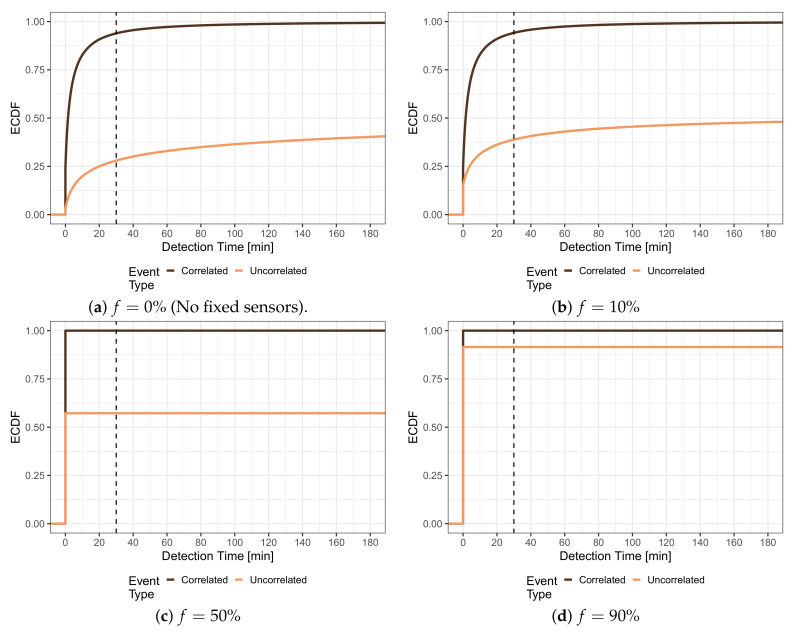
Distribution of detection time for uniform and population-based events in a hybrid system with fixed sensors. The fixed sensors are placed according to the relative density of people, i.e., they are placed in the *f*% of *visited* cells, which have the lowest density of people.

To improve the detection of correlated events that are distributed according to the density of people, it is not beneficial to place fixed sensors in cells that people never visit because no correlated events can appear there; thus, Figure 9 depicts an investigation of hybrid detection systems, which *place the fixed sensors according to the relative density of people*. This means fixed sensors are first distributed to the *visited* cells that have the lowest density of people. After all visited cells are equipped, i.e., for f>42%, fixed sensors are also placed randomly in vacant cells.

Again, Figure 9a shows the baseline distributions of detection time of correlated (dark curve) and uncorrelated (light curve) events as a reference, cf. Figure 3. Figure 9b presents the results for a system with fixed sensors in f=10% of the cells. It can be seen that the detection of uncorrelated events (light curve) is improved with shorter detection times. The distribution of the detection times for uncorrelated events is very similar to Figure 8b, as for uncorrelated events, only the increase in coverage by the fixed sensors affects the detection performance regardless of their position. The detection of correlated events (dark curve) is not significantly affected by the additional fixed sensors, and the share of immediately detected uncorrelated events increases only marginally from 24.18% to 24.46%. When the share of fixed sensors increases to f=50% in Figure 9c, almost all populated cells (51%) are covered with fixed sensors. It can be seen that all correlated events (dark curve) are immediately detected because they only appear in populated cells. Further, 57.33% of the uncorrelated events (light curve) can be immediately detected in the investigated simulation run. As the other cells are vacant, mobile sensors cannot increase the detection performance in this case, which can also be seen from the horizontal shape of the orange CDF. Finally, Figure 9d shows the detection time distributions for a share of f=90%. As expected, the share of detected uncorrelated events has increased to 91.46%, and all events are either detected immediately or not at all.

The results show that a hybrid system with additional fixed sensors can successfully improve the detection performance for both uncorrelated and correlated events; however, depending on the type of events and the coverage of the mobile sensors, different placement strategies for fixed sensors have to be applied.

**Figure 10:**
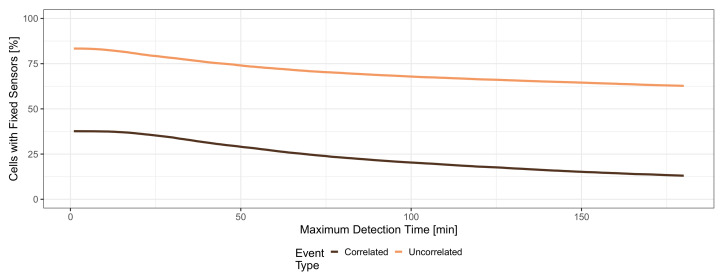
Needed share of cells with fixed sensors for keeping all detection times below a threshold.

In the following section, we describe our investigation into how many fixed sensors are needed in a hybrid MCS system to keep a certain detection time threshold for all events. This means no event should be missed, and all detection times should be shorter than a given maximum detection time threshold. Figure 10 shows the share of cells that have to be equipped with fixed sensors, on the y-axis for different maximum detection time thresholds from 0 to 180 min on the x-axis. The colored lines depict the results for correlated events (dark curve) and uncorrelated events (light curve). The presented results show a best-case analysis, i.e., the minimum share of cells with a fixed sensor. Thereby, an optimal placement of fixed sensors was assumed, which considers the appearance of all future events and the movement of mobile sensors in the simulation run. In a real system, the appearance of future events and the movement of mobile sensors cannot be accurately predicted to obtain an optimal placement, and thus, more cells might have to be equipped with fixed sensors to keep a given maximum detection threshold. Further, the budget for buying, installing, operating, and maintaining fixed sensors has to be considered and might further constrain the maximum detection threshold or the detection rate.

The light curve in Figure 10 shows the minimum share of cells that have to be equipped with a fixed sensor to keep the detection time for all uncorrelated events below a given maximum value. For an immediate detection of all events (maximum detection time is 0 min), at least 83.35% of the cells have to be equipped with a fixed sensor. In the remaining cells, all appearing events were immediately detected by MCS because a mobile sensor was present during the appearance of the event. We observe that fewer fixed sensors are needed for higher detection time thresholds because MCS can detect more events within the given detection time. For detecting all events within 30 min at least 78.15% of cells have to be equipped. If the threshold is set to 180 min, fixed sensors have to be placed in only at least 62.72% of the cells.

When considering correlated events (dark curve in Figure 10), generally fewer cells have to be equipped with fixed sensors because the events can only appear in visited cells. Thus, fixed sensors have to be placed only in at least 37.66% of all cells to detect all events immediately. Again, this number decreases when the maximum detection time threshold is relaxed. Only at least 34.20% of the cells have to be equipped for holding a threshold of 30 min, while only at least 13.05% of the cells need a fixed sensor to keep a threshold of 180 min. This need for fixed sensors in visited cells even for such high detection time thresholds might seem counter-intuitive compared to the results for uncorrelated events; however, it has to be remembered that the location of correlated events does not follow a uniform distribution truncated to visited cells only, but it has a completely different distribution identical to the density of people. Thus, fixed sensors are still needed for cells that are visited, but in which mobile sensors move very rarely. If events appear in such a cell, they might face long detection times until a mobile sensor moves there, which have to be shortened by placing fixed sensors.

The results show that fixed sensors are still needed to keep maximum detection time thresholds for MCS systems; however, they do not have to be placed in all cells, which means that adding mobile MCS can reduce the need for fixed sensors. As the appearance of events and the movement of mobile sensors cannot be predicted in practice, still a higher amount of fixed sensors might be needed than presented in Figure 10, where an optimal placement was assumed. Further, in practice, the budget has to be considered as it limits the number of fixed sensors. Thus, there will always be a trade-off between the maximum detection time threshold and the detection rate, which depends not only on the mobile MCS but also on the actual placement of the fixed sensors.

## 6. Discussion

In this work, we conducted a simulative performance evaluation of MCS to investigate trade-offs for the event detection scenario. For this, we prepared a mobility trace of the city of Würzburg with random walks of pedestrians over the course of a single day corresponding to an MCS participation of roughly 1% of the population. Events were simulated to appear uniformly random during 24 h, and their locations were randomly spread over the map of Würzburg. For this, we considered three types of event location distributions, namely, correlated to the population density, partially correlated, or uniformly random over the entire map (uncorrelated). It was shown that the trade-offs between detection time and detection probabilities could be adjusted by different maximum detection time thresholds, although this threshold might be determined by the type of detected event in practice. For the remaining analyses, we assumed a maximum detection time of 30 min, which showed a good compromise between low detection times and high detection probabilities.

The evaluation showed that correlated events faced long detection times and a rather low detection rate of 27.98% (detection threshold 30 min). This is mainly caused by areas on the map that have no roads, and thus, are never visited by mobile sensors. Thus, events appearing in these cells cannot be detected by MCS. The detection rate could be increased up to 40.27% by setting a higher maximum detection time of 180 min; however, with such high detection time threshold, in practice, events might disappear or become irrelevant in the meantime. Considering correlated events, the evaluation showed generally much shorter detection times and high detection rates of 93.91% within 30 min and 99.93% within 180 min. High detection rates could even be observed during the night. The detection of correlated events was also much less sensitive to setting a maximum detection time than the detection of uncorrelated events. As partially correlated events consisted of *p*% correlated and (100−p)% uncorrelated events, the results for a given percentage of *p* can be interpolated from both marginal cases.

The performance evaluation showed that MCS could achieve almost total coverage of the city for correlated events. Moreover, it was confirmed that detection rates for all kinds of events could be increased by increasing participation in MCS. Although it was found that most detected events could be detected very fast, the distribution showed a long tail. This means that a considerable amount of events faced significant detection times or could not be detected at all by MCS, especially for uncorrelated events. Thus, the performance evaluation confirmed the need to consider other means to increase the sensor coverage and decrease event detection time. Such means could be adding fixed sensors in a hybrid crowdsensing system or employing active crowdsensing, in which users are actively recruited and paid for sensing sparsely frequented or missing locations/times.

As a start, a hybrid system of MCS and additional fixed sensors was investigated. The results showed that equipping vacant cells, i.e., cells where no mobile sensors move, with fixed sensors can increase the overall detection rate for uncorrelated events. It does not affect the detection performance for correlated events that only appear where people move. When fixed sensors are only placed in cells that people visit, the detection performance for uncorrelated events increases almost by the same amount as if sensors are placed in vacant cells due to the uniformly distributed event location; however, the contribution of additional MCS is reduced when fixed sensors are placed in cells that mobile sensors can also cover. Placing fixed sensors in visited cells could only reduce the detection times for detecting correlated events because MCS almost achieved a perfect coverage.

The detection performance in hybrid systems largely depends on the placement strategy of the fixed sensors. Thereby, the characteristics of the event type and mobile sensors’ movement have to be taken into account. Considering an optimal placement, the minimal amount of fixed sensors was investigated that is needed to keep a certain detection time threshold. This means no event should be missed, and all events should be detected before the maximum detection time. It was found that an immediate detection of all events was possible for both uncorrelated and correlated events without placing fixed sensors in every cell. This shows that adding MCS is able to reduce the need for fixed sensors. When the maximum detection time threshold is relaxed, fewer cells need to be equipped with fixed sensors. Still, a practical placement of fixed sensors in a hybrid MCS system needs to consider not only the budget, the type of event, and the movement of mobile sensors but can also trade-off detection rate and maximum detection times. Further, the presented placement uses prior knowledge about the events and the distribution of the MCS participants that is not available in a real-world setting. Thus, the results show a best-case scenario and can be used as benchmarks for other placement or crowd-orchestration mechanisms, such as active crowdsensing.

In future work, the performance evaluation of the event detection scenario could be improved by considering more realistic mobility traces including vehicular mobility. Additionally, comparing real-world traffic and movement traces with the simulated SUMO traces would be of interest. Similarly, a study of different city traffic networks and topologies. Moreover, the impact of other distributions of event locations can be studied. The economic dimension in hybrid systems could be further evaluated, especially considering the trade-offs between needs and costs for fixed sensors. Further, the employment of active crowdsensing as an alternative or in addition to the placement of fixed sensors can be considered. One question in this context is whether the sensing budget should be better spent for fixed sensors with high reliability and longevity but low flexibility, or for the active recruitment of crowdworkers, which have high flexibility of data collection but might be less available or reliable. Finally, new scenarios, such as periodic sensing and continuous sensing, could be tackled.

## 7. Conclusions

This paper investigated the detection capabilities of hybrid systems, i.e., systems consisting of fixed sensors and mobile crowdsourcing users, concerning different event types. The results showed that hybrid systems could considerably improve detection time and probability compared to pure MCS, especially for events that appear uncorrelated to the population density; thus, hybrid crowdsensing can efficiently detect various event types in smart cities, even if only a small share of the population participates in the sensing efforts and only a relatively small number of sensors is deployed.
